# Low-Intensity Pulsed Ultrasound Stimulation Modulates the Nonlinear Dynamics of Local Field Potentials in Temporal Lobe Epilepsy

**DOI:** 10.3389/fnins.2019.00287

**Published:** 2019-04-02

**Authors:** Xin Li, Huifang Yang, Jiaqing Yan, Xingran Wang, Xiaoli Li, Yi Yuan

**Affiliations:** ^1^Institute of Electrical Engineering, Yanshan University, Qinhuangdao, China; ^2^College of Electrical and Control Engineering, North China University of Technology, Beijing, China; ^3^State Key Laboratory of Cognitive Neuroscience, Beijing Normal University, Beijing, China

**Keywords:** LIPUS, TLE, nonlinear dynamics, LFP, mice

## Abstract

Low-intensity pulsed ultrasound stimulation (LIPUS) can inhibit seizures associated with temporal lobe epilepsy (TLE), which is the most common epileptic syndrome in adults and accounts for more than half of the cases of intractable epilepsy. Electroencephalography (EEG) signal analysis is an important method for studying epilepsy. The nonlinear dynamics of epileptic EEG signals can be used as biomarkers for the prediction and diagnosis of epilepsy. However, how ultrasound modulates the nonlinear dynamic characteristics of EEG signals in TLE is still unclear. Here, we used low-intensity pulsed ultrasound to stimulate the CA3 region of kainite (KA)-induced TLE mice, simultaneously recorded local field potentials (LFP) in the stimulation regions before, during, and after LIPUS. The nonlinear characteristics, including complexity, approximate entropy of different frequency bands, and Lyapunov exponent of the LFP, were calculated. Compared with the control group, the experimental group showed that LIPUS inhibited TLE seizure and the complexity, approximate entropy of the delta (0.5–4 Hz) and theta (4–8 Hz) frequency bands, and Lyapunov exponent of the LFP significantly increased in response to ultrasound stimulation. The values before ultrasound stimulation were higher ∼1.87 (complexity), ∼1.39 (approximate entropy of delta frequency bands), ∼1.13 (approximate entropy of theta frequency bands) and ∼1.46 times (Lyapunov exponent) than that after ultrasound stimulation (*p* < 0.05). The above results demonstrated that LIPUS can alter nonlinear dynamic characteristics and provide a basis for the application of ultrasound stimulation in the treatment of epilepsy.

## Introduction

Epilepsy is one of the most common neurological diseases in the world. Repeated seizures will cause anxiety, depression, accidental disability, and even death and greatly affect quality of life (Viale et al., 2015; Bielefeld et al., 2017; O’Connell et al., 2017). The reason for epileptic seizures is the sudden abnormal firing of neurons. During epileptic seizures, there are transient dysfunctions in the brain that trigger short-term obstacles in patient movement and consciousness (Bjørk et al., 2015; Robertson et al., 2015; Xu et al., 2016). Lesions in different parts of the brain cause different types of epilepsy, and there is no one brain area associated with all types of epilepsy. Temporal lobe epilepsy (TLE) is the most common epileptic syndrome in adults and accounts for more than half of the cases of intractable epilepsy. Clinically, TLE has the characteristics of frequent seizures and difficult diagnosis and treatment (Monti and Meletti, 2015; Lévesque et al., 2016; Steinhäuser et al., 2016).

Electroencephalography (EEG) signals of epilepsy are an important tool for studying epilepsy (Mario et al., 2003; Noachtar and Rémi, 2009). Studies have shown that excessive repetitive firing of some neurons may cause their EEG time series to be in a lower dimensional chaotic motion state with epileptic seizure (Bosl et al., 2017). The characteristic parameters of EEG chaos can be used as biomarkers for the prediction and diagnosis of epilepsy (Kopczynska et al., 2018). Past studies have shown that complexity, approximate entropy and Lyapunov exponents were used as biomarkers of nonlinear characteristics of epileptic EEG signals (Varatharajah et al., 2018).

The complexity of EEG signals and functional status in the brain during epileptic seizures can be quantitatively evaluated by complexity (Mei et al., 2018). Previous studies have demonstrated that the complexity of EEG signals during epileptic seizures is lower than that in healthy humans (Wen-Chin et al., 2015; Noel et al., 2017). The complex characteristics and probability of chaotic systems can be reflected by the entropy. The epileptic seizures can be judged and measured by entropy as a biomarker (Li et al., 2018; Raghu et al., 2018). The sensitivity of the chaotic system to different initial values can be evaluated by the Lyapunov exponent. In the epileptic interval, the Lyapunov exponent is at a relatively low value; during the seizure, the Lyapunov exponent drops sharply and then gradually increases. After the seizure, the Lyapunov exponent remains elevated for a period of time and then returns to normal levels (Osowski et al., 2007). The above studies showed that the nonlinear characteristic parameters, including complexity, approximate entropy and Lyapunov exponent, changed significantly during epileptic seizures and can be used as biomarkers for the prediction and diagnosis of seizures.

Low-intensity pulsed ultrasound stimulation (LIPUS), as a new noninvasive neuromodulation technique, has received increasing attention (Landhuis, 2017). LIPUS has the advantage of high spatial resolution and high penetration depth (Alexander et al., 2011). The potential mechanisms of ultrasound stimulation for neuromodulation may be that ultrasound wave alter intramembrane cavitation, membrane conductance or mechanically sensitive ion channels to cause neuronal discharge (Tyler, 2011; Plaksin et al., 2014; Ye et al., 2018). Previous studies used LIPUS to stimulate different regions of the brain of rodent animals, monkeys, and humans. They found that LIPUS can modulate neuronal activity (Tyler et al., 2008; Tufail et al., 2010), neural network connections (Yu et al., 2016), cerebral hemodynamics (Yang et al., 2018; Yuan et al., 2018), and water molecular diffusion (Yuan et al., 2017). Previous studies have also shown that LIPUS can cause a reduction in the occurrence of epileptic EEG bursts and severity of epileptic behavior and can also result in fewer spontaneous recurrent seizures and improved performance in behavioral tasks assessing sociability and depression in the chronic period of epilepsy (Min et al., 2011; Hakimova et al., 2015). However, how LIPUS affects the key biomarkers of the nonlinear characteristics of EEG signals in TLE is still unclear.

To address this issue, we used LIPUS to stimulate the CA3 region of KA-induced TLE mice, simultaneously recorded local field potentials (LFP) in the stimulation regions before, during and after ultrasound stimulation. The nonlinear characteristics, LFP were analyzed.

## Materials and Methods

### Animals and Surgery

**FIGURE 1:**
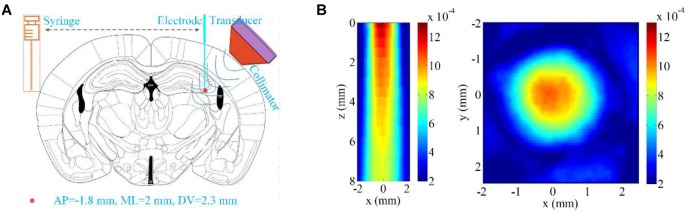
Ultrasound stimulation region and ultrasound field distribution. **(A)** The region for drug injection and LFP recording (AP = -1.8 mm, ML = 2 mm, DV = 2.3 mm relative to Bregma). **(B)** The two-dimensional ultrasound distribution in the *xz* and *xy* planes.

Fourteen C57BL/6 mice were randomly separated into two groups, the LIPUS group and the control group (all male, body weights 20–25 g, Beijing Vital River Laboratory Animal Technology Co., Ltd., China). Our study protocols were submitted to and approved by the Animal Ethics and Administrative Council of Yanshan University. All mice were anesthetized by 2% isoflurane (RWD Life Science Co., Shenzhen, China) in the experiment. A closed-loop animal temperature maintenance instrument (69002, Ruiwode, Shenzhen, China) was used to maintain body temperature at ∼37°C during all experiments. We referred to the previous work to make the TLE mouse model (Hakimova et al., 2015). As shown in Figure 1A, KA (1 mg/1 ml in saline, Tocris, United States) was unilaterally microinfused into the CA3 area (AP = -2.0 mm, ML = -2.3 mm, and DV = 2.0 mm from the bregma). Tungsten microelectrodes (WE50030.1B10, MicroProbe, United States) were inserted into the injection region 2 h after KA infusion.

### LIPUS Protocol and LFP Signal Recording

The LIPUS system was similar to that used in our previous paper (Yuan et al., 2018). In the LIPUS group, the fundamental frequency (FF), pulsed repetition frequency (PRF), duty cycle (DC), and stimulation duration (SD) of the pulsed ultrasound were 500 kHz, 500 Hz, 50% and 30 s, respectively. In the control group, the parameter settings were the same as those in the LIPUS group, excepted that we turned off the power amplifier. The unfocused ultrasound transducer (V301-SU, Olympus, United States) was connected to the mouse skull by a 3D printed conical collimator filled with ultrasound coupling gel. The diameter of the hole at the bottom of the conical collimator was 2 mm. The two-dimensional ultrasound distribution under *ex vivo* mouse skull in the *xz* and *xy* planes shown in Figure 1B was measured by a calibrated needle-type hydrophone (HNR500; Onda, United States) that was moved by a two-dimensional electric translation platform. The ultrasound pressure of both pulsed and continuous ultrasound was 0.26 MPa. A microelectrode inserted into the CA3 region was used for recording the LFP signals before, during and after LIPUS. The amplification and acquisition of LFP signals was the same as in our previous study (Yuan et al., 2016). Raw LFP signals were acquired at a sampling frequency of 2 kHz, and a low-pass filter with a 1000-Hz cutoff frequency was set in the differential preamplifier system.

The seizures were defined by the LFP according to the previous literature (amplitude: 3 × background, duration: > 10 s) (Hakimova et al., 2015). When seizures were confirmed, the LFPs were continuously recorded for 90 s in the control group. When the seizures lasted more than 30 s in the LIPUS group, we applied ultrasound stimulation for 30 s and recorded for a total duration of 90 s.

### Nonlinear Dynamics Analysis of LFP Signals and Statistical Analysis

#### Lemple-Zie Complexity

We calculated the Lemple-Zie complexity of the LFP according to previous literature (Sabeti, 2009). The normalized Lemple-Zie complexity can be expressed as

(1) LZC=C(n)/b(n)

where *C*(*n*) is the complexity of the LFP sequence, *b*(*n*) = *N*/log_2_(*n*). When *N* tends to infinity, *b*(*n*) provides an asymptotic value of *C*(*n*).

#### Approximate Entropy

We extracted the signals of different frequency bands (delta [0.5–4 Hz], theta [4–8 Hz], alpha [8–13 Hz], beta [13–30 Hz], gamma [30–45 Hz]) from the LFP and then calculated the approximate entropy of the signals in different frequency bands based on the literature (Jing et al., 2006; Mateo et al., 2006). The approximate entropy can be expressed by the following equation:

(2) $$\mathrm{ApEn}(m,\;r,\;N)=\phi^{m}(r)-\phi^{m+1}(r)$$

where *N* is the length of data, m is the vector dimension, and *r* is the tolerance.

#### Largest Lyapunov Exponent

The largest Lyapunov exponent was calculated by the method mentioned in a previous study (Osowski et al., 2007). The largest Lyapunov exponent can be expressed as

(3) $$L=\frac{1}{K\Delta t}\sum_{j=1}^{K}\log_{2}\frac{d_{j}(\Delta t)}{d_{j}(0)}$$

The detailed parameters of the above equation can be found in the literature (Osowski et al., 2007).

#### Statistical Analysis

The results were analyzed by the Kruskal–Wallis test, and a difference was deemed significant if *p* < 0.05. Statistical analysis was performed using MATLAB software.

## Results

### Effect of LIPUS on LFP Complexity in Acute TLE

**FIGURE 2:**
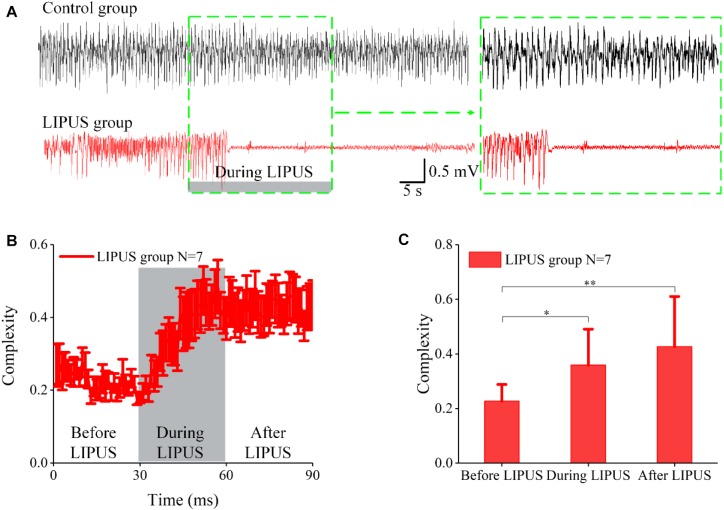
LFPs of epileptic seizures in both the control and LIPUS groups and the complexity analysis. **(A)** LFPs of epileptic seizures in control groups for 90 s (upper); LFPs of epileptic seizures in LIPUS groups for 90 s (lower). **(B)** The Lemple-Zie complexity of LFPs before, during and after ultrasound stimulation. **(C)** The mean values of complexity within 30 s before, during and after LIPUS (^∗^*p* < 0.05, ^∗∗^*p* < 0.01, *N* = 7, Kruskal–Wallis test).

We first proved that LIPUS can inhibit TLE seizures. Figure 2A(upper) shows continuous seizure for 90 s in the epilepsy group without LIPUS. In the LIPUS group [Figure 2A(lower)], continuous seizure lasted for 30 s. When ultrasound stimulation began, the epileptic seizure was not immediately suppressed, but was suppressed as the ultrasound stimulation continued and stopped after LIPUS. This result was consistent with that in the previous literature (Hakimova et al., 2015).

To evaluate the effect of LIPUS on LFP complexity in acute TLE, we calculated the Lemple-Zie complexity of LFPbefore, during and after ultrasound stimulation. As shown in Figure 2B, the complexity was at a low level and remained the same as that observed before LIPUS. When ultrasound stimulation was applied, the complexity gradually increased during LIPUS and was then elevated after LIPUS. We calculated the mean values of complexity within 30 s before, during and after LIPUS, and the results are shown in Figure 2C. The complexity after LIPUS was ∼1.87 times higher than that before LIPUS, representing a significant difference (^∗∗^*p* < 0.01, *N* = 7, Kruskal–Wallis test). In the control group, as shown in Supplementary Figures S1A,B, there was no significant change in complexity during the 90-s period. The above results indicated that ultrasound stimulation can inhibit TLE seizures and alter the complexity of epileptic LFP signals.

### Effect of LIPUS on the Approximate Entropy of the LFP in Acute TLE

**FIGURE 3:**
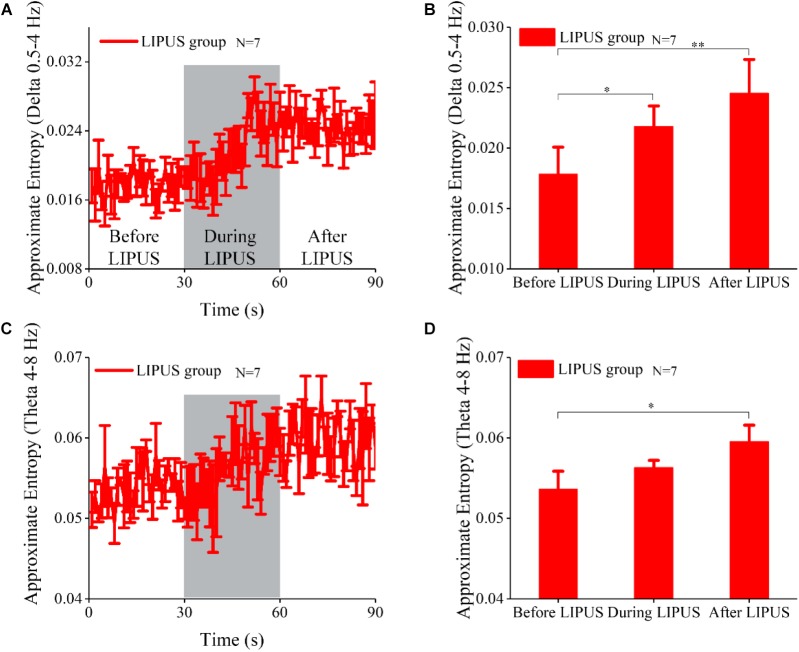
The approximate analysis of LFPs at different frequency bands. **(A)** The approximate entropy analysis of LFPs at the delta (0.5–4 Hz) frequency band before, during and after ultrasound stimulation. **(B)** The mean values of approximate entropy at the delta (0.5–4 Hz) frequency band within 30 s before, during and after LIPUS (^∗^*p* < 0.05, ^∗∗^*p* < 0.01, *N* = 7, Kruskal–Wallis test). **(C)** The approximate entropy analysis of LFPs at the theta (4–8 Hz) frequency band before, during and after ultrasound stimulation. **(D)** The mean values of approximate entropy at the theta (4–8 Hz) frequency band within 30 s before, during and after LIPUS (^∗^*p* < 0.05, *N* = 7, Kruskal–Wallis test).

The approximate entropy of different frequency bands is often used to evaluate changes in EEG signals. Here, we analyzed the approximate entropy of LFP signals at different frequency bands in the LIPUS group. As shown in Figure 3, the approximate entropy at the delta (0.5–4 Hz) and theta (4–8 Hz) frequency bands was significantly modulated by ultrasound. The trends of the changes in approximate entropy at the delta (0.5–4 Hz) and theta (4–8 Hz) frequency bands were similar to that of complexity. We also calculated the mean value of approximate entropy within 30 s before, during and after LIPUS (Figure 3B,D). The approximate entropy at delta and theta after LIPUS was ∼1.39 (delta) and 1.13 (theta) times higher than that before LIPUS (*N* = 7, Kruskal–Wallis test, ^∗^*p* < 0.05). As shown in Supplementary Figure S2, there was no significant change in approximate entropy at the frequency bands of alpha (8–13 Hz), beta (13–30 Hz), or gamma (30–45 Hz) before, during and after LIPUS. The approximate entropy remained at a stable level with epileptic seizures during the 90-s period in the control group, as shown in Supplementary Figure S3. The above results indicated that ultrasound stimulation can only modulate the approximate entropy of the LFP at the delta and theta frequency bands.

### Effect of LIPUS on the Largest Lyapunov Exponent of the LFP in Acute TLE

**FIGURE 4:**
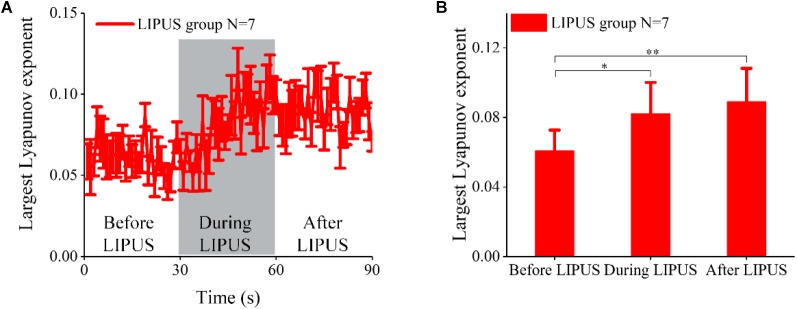
The Lyapunov exponent of LFPs in the LIPUS group. **(A)** The largest Lyapunov exponent analysis of LFPs before, during and after ultrasound stimulation. **(B)** The mean values of the largest Lyapunov exponent within 30 s before, during and after LIPUS (^∗^*p* < 0.05, ^∗∗^*p* < 0.01, *N* = 7, Kruskal–Wallis test).

Here, we analyzed the maximum Lyapunov exponent of the LFP in both the control and LIPUS groups. The experimental results of the LIPUS group were shown in Figure 4. The largest Lyapunov exponent did not change significantly with epileptic seizure and gradually increased with ultrasound stimulation, finally reaching a higher level after LIPUS. Statistical analysis indicated that the largest Lyapunov exponent after LIPUS was ∼1.46 times higher than that before LIPUS (*N* = 7, Kruskal–Wallis test, ^∗^*p* < 0.05). The Lyapunov exponent in the control group showed no significant change, as shown in Supplementary Figure S4. The above results verified that ultrasound stimulation can significantly modulate the maximum Lyapunov exponent of the LFP in acute TLE.

## Discussion

Electroencephalography signals contain a large amount of physiological and pathological information, and analysis of EEG signals is an important and powerful tool for studying epilepsy (Acharya et al., 2011). In past studies, researchers have proposed many methods to evaluate the diagnostic and therapeutic effects of epilepsy by analyzing epileptic EEG signals (Giannakakis et al., 2014). In these identification methods for epilepsy, the nonlinear characteristic parameters of EEG, including entropy, complexity and Lyapunov exponent, are often used as important biomarkers.

In this study, low-intensity ultrasound was used to stimulate the CA3 region for acute TLE, and LFPs of the CA3 region were simultaneously recorded. Our results demonstrated that low-intensity ultrasound can effectively modulate nonlinear dynamics in TLE. The difference in the nonlinear characteristics of epileptic EEGs is closely related to the abnormal synchronous firing of brain neurons during seizures (Lehnertz, 2008). Ultrasound stimulation modulates the abnormal synchronous firing of brain neurons and inhibits epileptic seizures, thus causing a significant change in the nonlinear dynamic characteristics of epileptic EEGs. To our knowledge, this is the first report on the nonlinear dynamic characteristic of ultrasound modulation of seizures in acute TLE.

Epilepsy EEG analysis has an important guiding role in the treatment of epilepsy by LIPUS. In this study, we demonstrated that LIPUS can modulate the nonlinear dynamic characteristics of epilepsy EEG. Therefore, when LIPUS is used to intervene in the electrophysiological activity of the brain, if the nonlinear dynamic characteristics of the EEG signal can be monitored, the different states of epilepsy can be correctly identified. Furthermore, this technique can guide the selection of appropriate ultrasound parameters, such as ultrasound intensity, pulsed repetition frequency, stimulation duration or duty cycles.

In the previous study, Hakimova et al. used low-intensity ultrasound to stimulate mice with TLE and found that LIPUS can effectively inhibit seizure of TLE. There are some similarities and differences between our and Hakimova et al’s studies. (1) Similarities: KA was used to make mouse model of TLE and the location of the KA injection and recording electrode was the same. The duration of ultrasound stimulation was 30 s in both studies. (2) Differences: In Hakimova et al’s study, they investigated behavior, number of seizures, latency of seizure and seizure spikes of mice with TLE under LIPUS. In our study, we investigated the nonlinear characteristics including complexity, approximate entropy of different frequency bands, and Lyapunov exponent of the LFPs from CA3 region of mice with TLE under ultrasound stimulation.

In humans, there is significant pyramidal cell loss in the hippocampal subfields CA1 and CA3 (DeGiorgio et al., 1992). Because ultrasound stimulation with single parameter has no specific selectivity to neurons, we speculate that it can also cause changes of nonlinear characteristics of LFPs in CA1 region of TLE mice.

There are two limitations and challenges to our study: (i) ultrasound stimulation can inhibit acute TLE seizures and modulate the nonlinear dynamics characteristic of LFPs. However, the accurate molecular mechanism by which ultrasound stimulation inhibits epileptic seizures is still unknown. Low-intensity ultrasound stimulation can enhance the expression of brain-derived neurotrophic factors in astrocytes through activation of TrkB-Akt and Calcium-CaMK signaling pathways (Liu et al., 2017). We hypothesize that ultrasound modulates the expression of epileptic seizure-associated proteins, which may be the molecular mechanism by which ultrasound inhibits epileptic seizures, but this hypothesis needs to be verified in future work. (ii) In addition, previous studies have shown that ultrasonic parameters can cause different modulatory effects on neural activity (King et al., 2013; Yuan et al., 2016). We only studied the inhibition of single parameters in acute TLE. To find the optimal parameters for ultrasound inhibition of TLE, we will conduct multiparameter studies and identify the ultrasound parameters with the best therapeutic effect.

In summary, our study indicates that ultrasound stimulation can modulate the nonlinear dynamic characteristics of the LFP in acute TLE.

## Author Contributions

YY designed and coordinated the study. All authors carried out experiment and data process, drafted the manuscript, and gave final approval for publication.

## Conflict of Interest Statement

The authors declare that the research was conducted in the absence of any commercial or financial relationships that could be construed as a potential conflict of interest.
