# Supplementary material for: Low-Intensity Pulsed Ultrasound Stimulation Modulates the Nonlinear Dynamics of Local Field Potentials in Temporal Lobe Epilepsy
Source: Front Neurosci. 2019 Apr 2;13:287. doi: 10.3389/fnins.2019.00287 (PMC6454000; doi:10.3389/fnins.2019.00287)
Supplement: Supplementary file 1 [file Table_1.DOC]

**Low-intensity pulsed ultrasound stimulation modulates the nonlinear dynamics of local field potentials in temporal lobe epilepsy**

Xin Li^1^, Huifang Yang^1^, Jiaqing Yan^2^, Xingrang Wang^1^, Xiaoli Li^3*^ and Yi Yuan^1*^

^1^ Institute of Electrical Engineering, Yanshan University, Qinhuangdao, 066004, China

^2^ College of Electrical and Control Engineering, North China University of Technology, Beijing, 10041, China

^3^ State Key Laboratory of Cognitive Neuroscience, Beijing Normal University, Beijing, 100875, China

*Address correspondence to Xiaoli Li, Email: [xiaoli@bnu.edu.cn](mailto:xiaoli@bnu.edu.cn), Yi Yuan, Email: [yuanyi513@ysu.edu.cn](mailto:yuanyi513@ysu.edu.cn)

Supplementary materials:


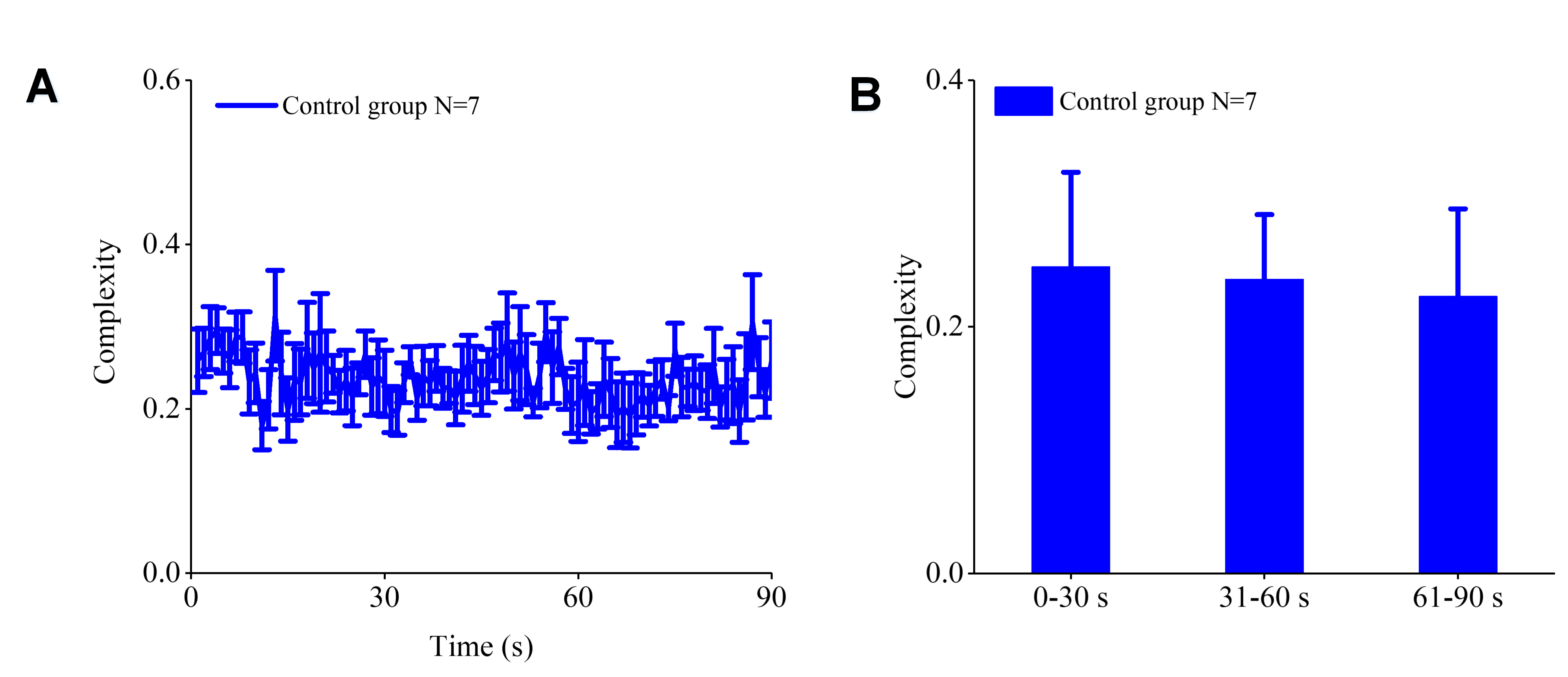


**Figure S1.** (A) The Lemple-Zie complexity of LFPs before, during and after ultrasound stimulation in the control group. (B) The mean values of complexity within 30 s before, during and after LIPUS in the control group.


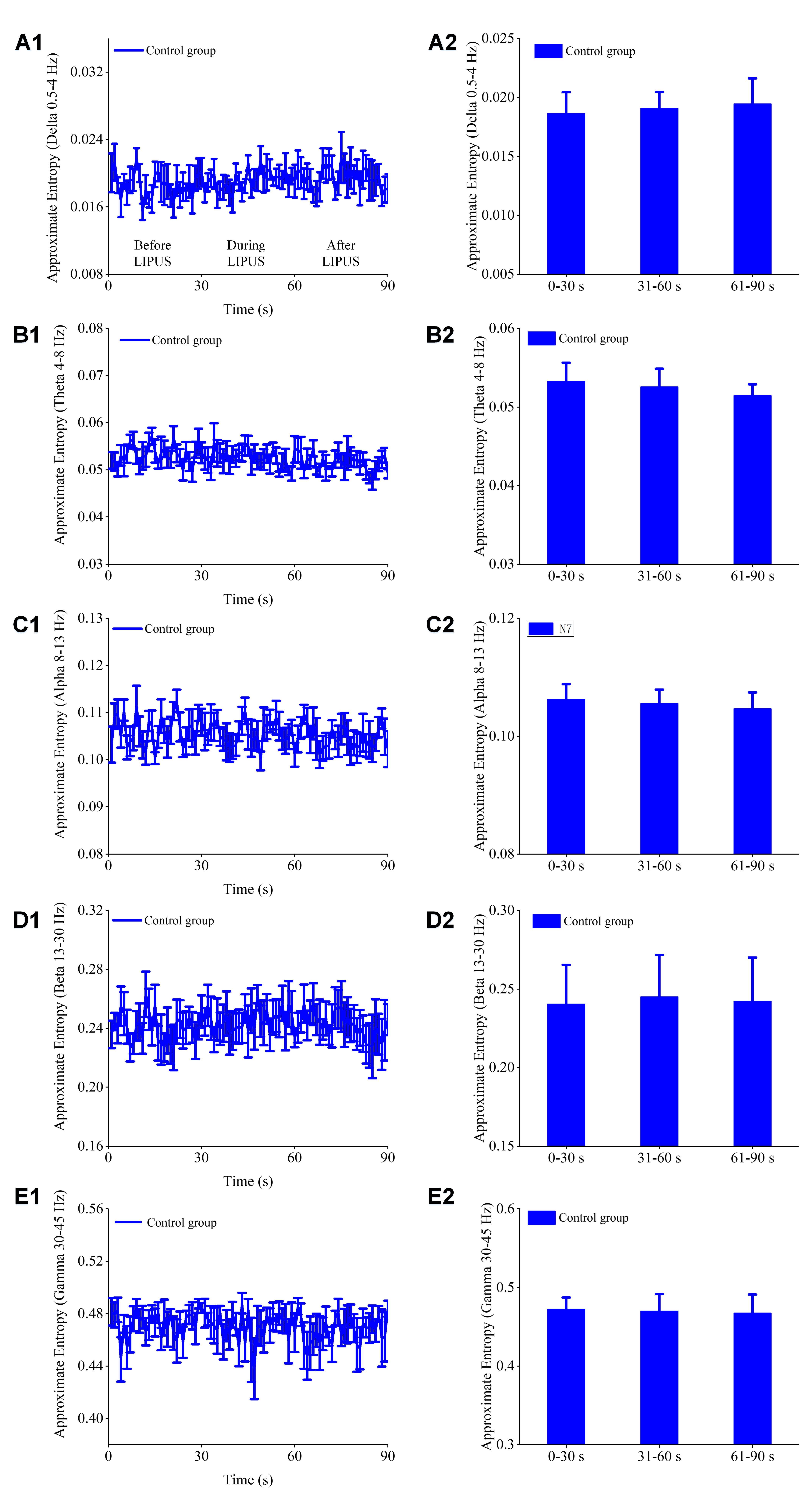


**Figure S2.** (A1-E1) The approximate entropy analysis of LFPs at different frequency bands ((delta [0.5-4 Hz], theta [4-8 Hz], alpha [8-13 Hz], beta [13-30 Hz], gamma [30-45 Hz])) before, during and after ultrasound stimulation in control group. (B2-E2) The mean values of approximate entropy at different frequency bands (delta [0.5-4 Hz], theta [4-8 Hz], alpha [8-13 Hz], beta [13-30 Hz], gamma [30-45 Hz]) before, during and after ultrasound stimulation in the control group.


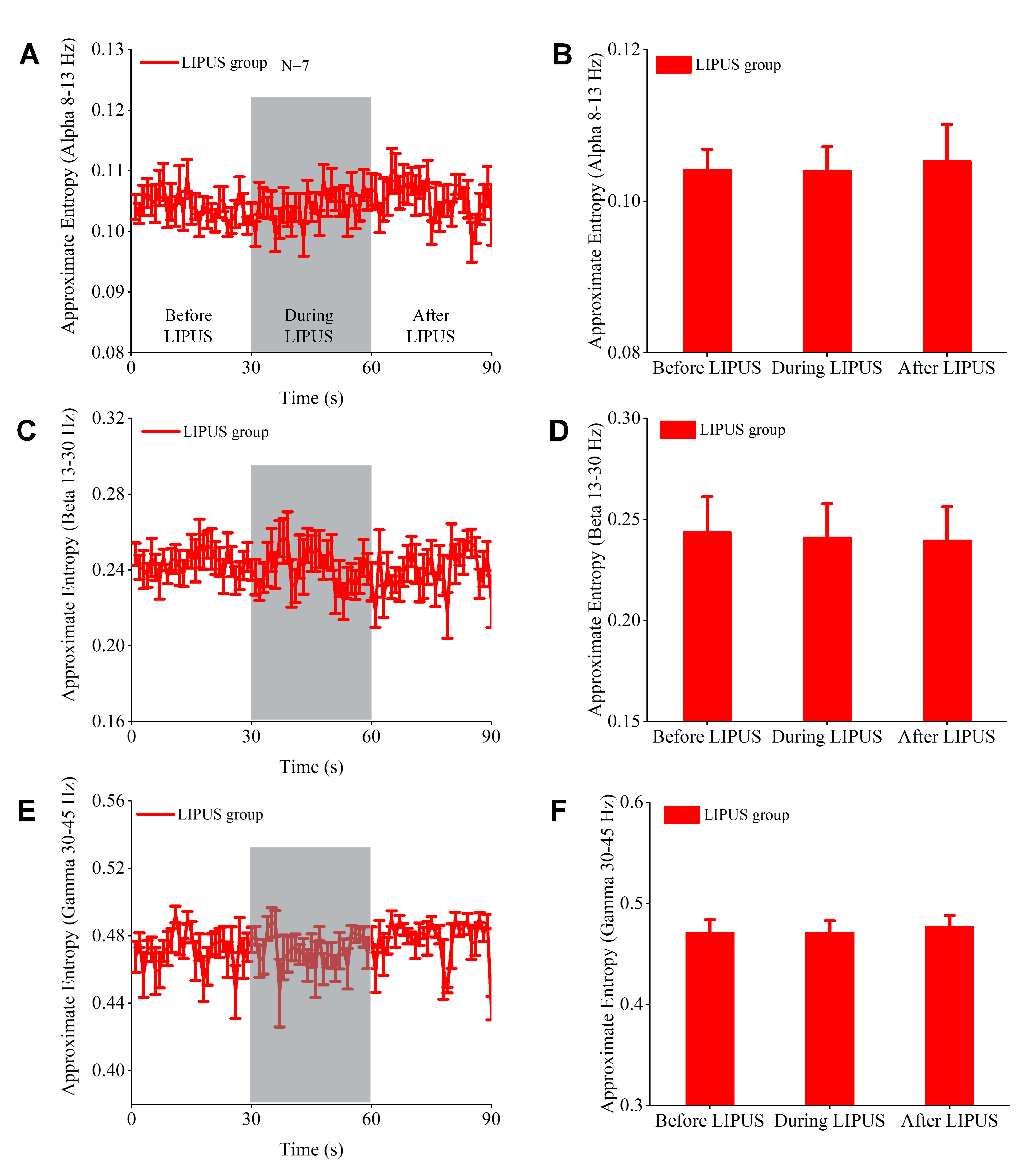


**Figure S3.** (A, C, E) The approximate entropy analysis of LFPs at different frequency bands (alpha [8-13 Hz], beta [13-30 Hz], gamma [30-45 Hz]) before, during and after ultrasound stimulation in the LIPUS group. (B, D, F) The mean values of approximate entropy at different frequency bands (alpha [8-13 Hz], beta [13-30 Hz], gamma [30-45 Hz]) before, during and after ultrasound stimulation in the LIPUS group.


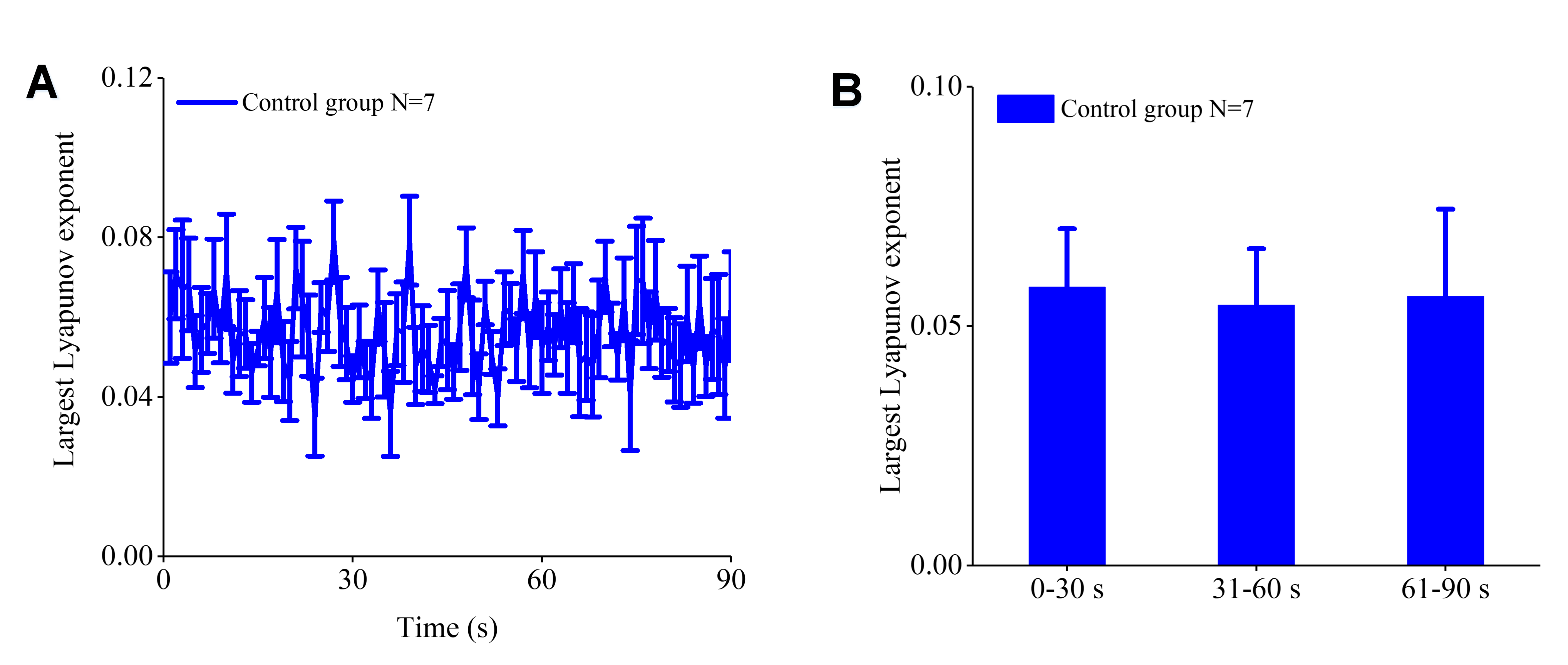


**Figure S4.** (A) The largest Lyapunov exponent analysis of LFPs before, during and after ultrasound stimulation in the control group. (B) The mean values of largest Lyapunov exponent within 30 s before, during and after ultrasound stimulation in the control group.
